# Global Burden of Thyroid Cancer in Children and Adolescents, 1990–2021: Trends, Disparities, and Future Projections

**DOI:** 10.3390/cancers17050892

**Published:** 2025-03-05

**Authors:** Tianyu Li, Zhen Cao, Chen Lin, Weibin Wang

**Affiliations:** Department of General Surgery, Peking Union Medical College Hospital, Chinese Academy of Medical Sciences and Peking Union Medical College, Beijing 100730, China; m17685768911@163.com (T.L.); caozhen1018@126.com (Z.C.)

**Keywords:** thyroid cancer, children and adolescents, incidence, mortality, projection

## Abstract

Background: Thyroid cancer is a rising concern in children and adolescents, with unique biological behaviors compared to adults. This study aimed to explore the epidemiological trends, pathological features, and regional disparities of thyroid cancer in this population using data from the Global Burden of Disease (GBD) 2021. Methods: Data on thyroid cancer incidence and mortality from 1990 to 2021 were extracted for individuals under 20 years old. The estimated annual percentage change (EAPC) was calculated to evaluate temporal trends. The Sociodemographic Index (SDI) was applied to assess regional variations. Future trends were projected using a Bayesian age–period–cohort model. Results: From 1990 to 2021, the global incidence of thyroid cancer in children and adolescents increased significantly, with an EAPC of 1.17%. Low-SDI regions exhibited the highest rise in incidence (EAPC: 2.19%), while high-SDI regions experienced a slight decline (EAPC: −0.69%). Mortality decreased globally (EAPC: −0.27%), with notable reductions in high- and middle-SDI regions but stable or increasing rates in low-SDI regions. Females consistently exhibited higher incidence rates across all SDI levels, while males in high-SDI regions showed higher mortality rates. Future projections suggest a steady decline in incidence and mortality rates through 2050. Conclusions: The increasing incidence and persistent mortality disparities of thyroid cancer in children and adolescents highlight the need for targeted public health interventions. Regions with low socioeconomic development require prioritized strategies to address this growing burden. These findings provide crucial insights for early diagnosis, treatment optimization, and global health policy formulation.

## Simple Summary

Thyroid cancer in children and adolescents is an increasingly significant concern, characterized by unique features that distinguish it from thyroid cancer in adults. This study examines global trends in thyroid cancer from 1990 to 2021, with a particular focus on disparities across regions with varying levels of economic development. This research aims to elucidate the evolving trends in thyroid cancer incidence across different regions and to project future trends up to 2050. By analyzing data on incidence and mortality rates, this study underscores the necessity for targeted public health strategies, particularly in underdeveloped regions with limited healthcare resources. The findings have the potential to enhance early detection, treatment, and awareness, ultimately reducing the global burden of thyroid cancer among young populations.

## 1. Introduction

Thyroid cancer is one of the most common endocrine malignancies, with its global incidence rising steadily over the past decades [1]. While much of the existing research focuses on adults, thyroid cancer in children and adolescents exhibits distinct biological behaviors and disease characteristics, necessitating specialized attention [2,3]. This younger population, defined as individuals under 20 years of age by the World Health Organization, faces unique challenges in terms of pathophysiology, clinical presentation, and long-term prognosis. For instance, compared to adult cases, pediatric thyroid cancer is more likely to present with larger tumors, increased local invasion, and a higher propensity for both cervical lymph node and distant metastases, particularly in younger children (≤10 years old) [4,5].

Despite these aggressive features, clinical attention to thyroid cancer in children and adolescents remains insufficient, with misconceptions surrounding its management. Papillary thyroid carcinoma (PTC) is the predominant histological subtype in this population, yet its unique progression patterns often go unrecognized. Moreover, external factors such as radiation exposure play a pivotal role in pediatric thyroid carcinogenesis, with studies indicating that even low-dose radiation (0.50 Gy) significantly increases the risk of thyroid nodules and cancer [6]. These findings underscore the importance of targeted research to inform clinical practice and improve outcomes.

Globally, the burden of thyroid cancer varies significantly across regions, reflecting disparities in socioeconomic development, healthcare access, and diagnostic capabilities. The Sociodemographic Index (SDI), a composite measure of income, education, and fertility, provides a critical framework for analyzing these disparities [7]. High-SDI regions report stable or declining mortality rates due to advanced healthcare systems, while low-SDI regions face increasing incidence and mortality trends, highlighting the urgent need for tailored interventions in resource-limited settings.

To address these challenges, this study utilizes data from the Global Burden of Disease (GBD) 2021 study to provide a comprehensive analysis of thyroid cancer in children and adolescents from 1990 to 2021, examining its incidence, mortality, and associated disparities across SDI levels. Furthermore, we applied a Bayesian age–period–cohort model to project future trends through 2050. By identifying sociodemographic disparities and unique disease characteristics, this study aims to fill critical knowledge gaps and support evidence-based strategies to reduce the global burden of thyroid cancer in young populations.

## 2. Methods

### 2.1. Overview and Data Collection

The GBD 2021 study analyzed the incidence and mortality of thyroid cancer across 204 countries and regions from 1990 to 2021 [8]. All of these regions have long-term data coverage, ensuring the reliability of the estimates derived from these regions. This annually updated global initiative organizes data by demographic factors such as age, sex, and the Sociodemographic Index (SDI). Using the Global Health Data Exchange platform (https://vizhub.healthdata.org/gbd-results/, accessed on 10 January 2025), we obtained standardized definitions, prevalence measures, and other essential metrics related to thyroid cancer. This study specifically focused on thyroid cancer in individuals under 20 years of age, who were categorized as children and adolescents. This categorization aligns with widely accepted definitions used by the World Health Organization, which defines adolescents as individuals aged 10–19 years old and children as those under 10 years old.

The GBD 2021 study uses the DisMod-MR 2.1 Bayesian meta-regression framework to combine data from various sources, adjusting for systematic biases to ensure the consistency of estimates for incidence and mortality [9]. Uncertainty intervals (UIs) are computed for all estimates to account for variability and provide an indication of confidence in the data [10].

### 2.2. Calculation of Estimated Annual Percentage Change (EAPC)

The EAPC is computed by applying a linear regression model to the natural logarithm of age-standardized rates (ASRs) across a defined time period [11,12]. First, the ASRs are log-transformed, and then a linear regression is performed with time (in years) as the independent variable and the log-transformed ASRs as the dependent variable. The slope (β) obtained from this regression represents the EAPC, which reflects the average annual percentage change in the ASRs. The formula used for calculation is EAPC = 100 × (exp(β) − 1), where β is the slope, and exp(β) refers to the exponentiation of β [13].

### 2.3. Sociodemographic Index

The Sociodemographic Index (SDI) measures the developmental status of regions or countries by incorporating fertility rates, income per capita, and educational levels [14]. The SDI ranges between 0 and 1, with higher values signifying more developed socioeconomic conditions. In the GBD 2021 dataset, countries and territories are classified into five SDI groups: high (>0.81), high–middle (0.70–0.81), middle (0.61–0.69), low–middle (0.46–0.60), and low (<0.46) [15].

### 2.4. Case Definition

In this research, we examined the incidence, mortality, and overall burden of thyroid cancer using data from the Global Burden of Disease study 2021. Thyroid cancer classification follows the guidelines of the Tenth Edition of International Classification of Diseases (ICD-10). Thyroid cancer is specifically coded under C73, representing malignant neoplasm of the thyroid gland. This code encompasses all anatomical and clinical presentations of thyroid cancer without further subcategories for specific sites or types.

### 2.5. Statistical Analysis

The burden of thyroid cancer was evaluated through incidence, DALYs, mortality, and their ASRs per 100,000 population. Projections for 2050 were made using a Bayesian age–period–cohort (BAPC) model [16,17]. This model employs integrated nested Laplace approximations (INLAs) for Bayesian inference, ensuring accurate trend forecasting [18,19]. Statistical analysis was conducted using R (version 4.2.1) software, with a two-sided *p*-value < 0.05 deemed statistically significant.

## 3. Result

### 3.1. Incidence and Temporal Trend

Globally and regionally, the incidence of thyroid cancer in children and adolescents increased significantly from 1990 to 2021 (Table 1). The global number of cases rose from 2981.4 (95% uncertainty interval: 2646.6–3405.2) in 1990 to 4906.0 (3999.1–6227.1) in 2021, with the incidence rate per 100,000 population increasing from 0.132 (0.117–0.151) to 0.186 (0.152–0.236). The estimated annual percentage change (EAPC) was 1.17%. Regions with a low Sociodemographic Index (SDI) showed the highest increase, with an EAPC of 2.19%, while high-SDI regions experienced a slight decline in both case numbers and the incidence rate (EAPC: −0.69%) (Table 1).

**Figure 1:**
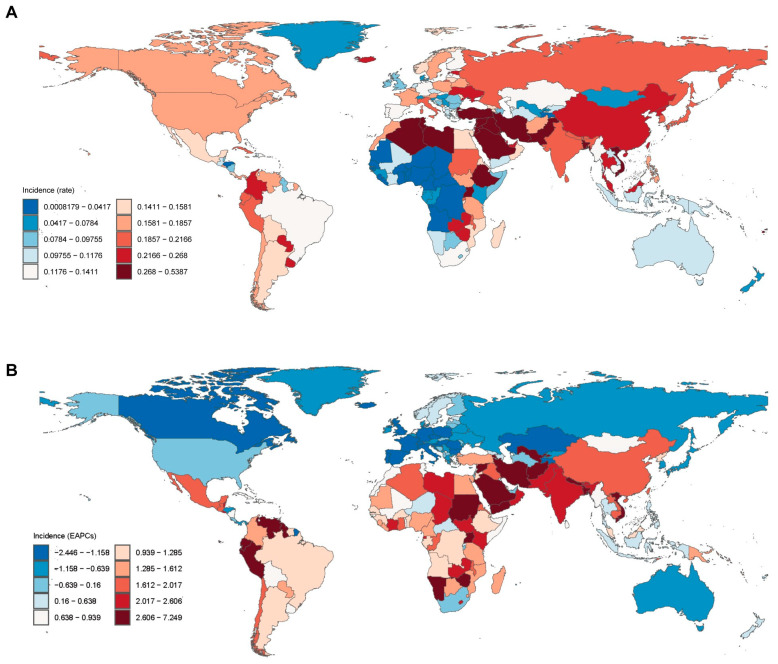
Global incidence rate (**A**) and estimated annual percentage change (EAPC) (**B**) in thyroid cancer in children and adolescents. Reprinted/adapted with permission from Ref. [20]. 2024, Institute for Health Metrics and Evaluation.

In 2021, the highest incidence rates were observed in high-income Asia Pacific (0.214 per 100,000 population), Western Europe (0.139), and high-income North America (0.181) (Figure 1A). These regions, however, also exhibited either minimal growth or declining trends, with EAPCs of −1.08%, −1.53%, and −0.32%, respectively. Conversely, regions with the most substantial increases in the EAPC, such as South Asia (2.74%), Andean Latin America (2.49%), and North Africa and the Middle East (2.17%), reported comparatively lower incidence rates of 0.240, 0.188, and 0.245, respectively (Figure 1A). Despite having lower incidence rates, regions like Eastern Sub-Saharan Africa (0.228, EAPC: 1.42%) and Southeast Asia (0.178, EAPC: 1.29%) exhibited notable upward trends.

At the regional level, South Asia and Andean Latin America exhibited the most pronounced growth, with EAPCs of 2.74% and 2.49%, respectively. In contrast, high-income Asia Pacific and Western Europe reported declines, with EAPCs of −1.08% and −1.53%, respectively (Figure 1B). Additionally, regions such as East Asia and Eastern Sub-Saharan Africa, despite having lower baseline incidence rates, demonstrated substantial increases over time.

### 3.2. Death and Temporal Trend

Globally, the mortality of thyroid cancer in children and adolescents decreased slightly from 1990 to 2021. The number of deaths increased from 348.8 (95% uncertainty interval: 305.5–405.9) in 1990 to 371.6 (294.2–479.6) in 2021, while the death rate per 100,000 population decreased from 0.015 (0.014–0.018) to 0.014 (0.011–0.018). The estimated annual percentage change (EAPC) was −0.27% (Table 2).

**Figure 2:**
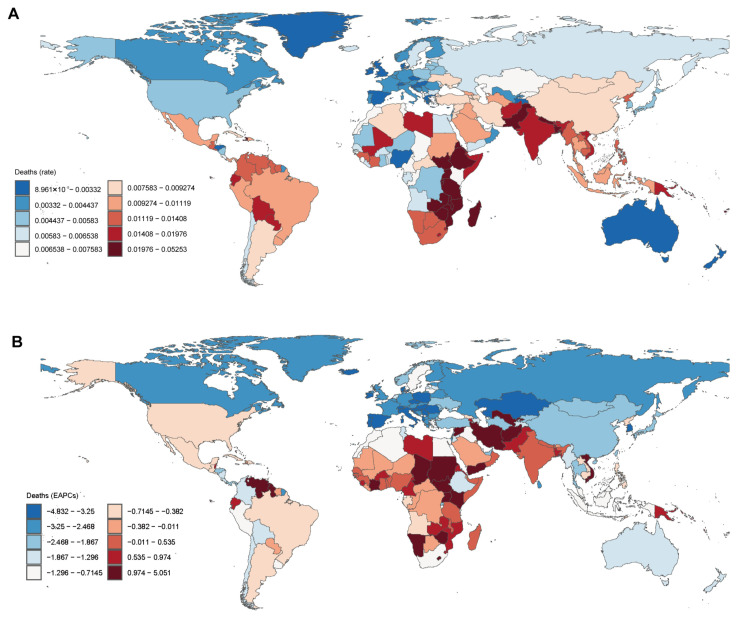
Global death rate (**A**) and estimated annual percentage change (EAPC) (**B**) in thyroid cancer in children and adolescents. Reprinted/adapted with permission from Ref. [20]. 2024, Institute for Health Metrics and Evaluation.

In 2021, the highest death rates were observed in South Asia at 0.024 per 100,000 population, followed by Eastern Sub-Saharan Africa at 0.032 and Southern Sub-Saharan Africa at 0.018 (Figure 2A). The lowest death rates were reported in Australasia at 0.003, high-income North America at 0.004, and Western Europe at 0.003 (Figure 2A).

The largest declines in the EAPC were observed in Central Europe (−4.28%), Eastern Europe (−2.77%), and East Asia (−2.30%), indicating significant reductions in mortality. Other regions with notable decreases included high-income Asia Pacific (−2.70%) and Western Europe (−3.13%) (Figure 2B). In contrast, positive EAPCs were recorded in Southern Sub-Saharan Africa (0.77%), South Asia (0.60%), and Oceania (0.30%). Western Sub-Saharan Africa also showed a smaller positive EAPC of 0.28%, reflecting an increasing trend in mortality over the three decades (Figure 2B).

**Figure 3:**
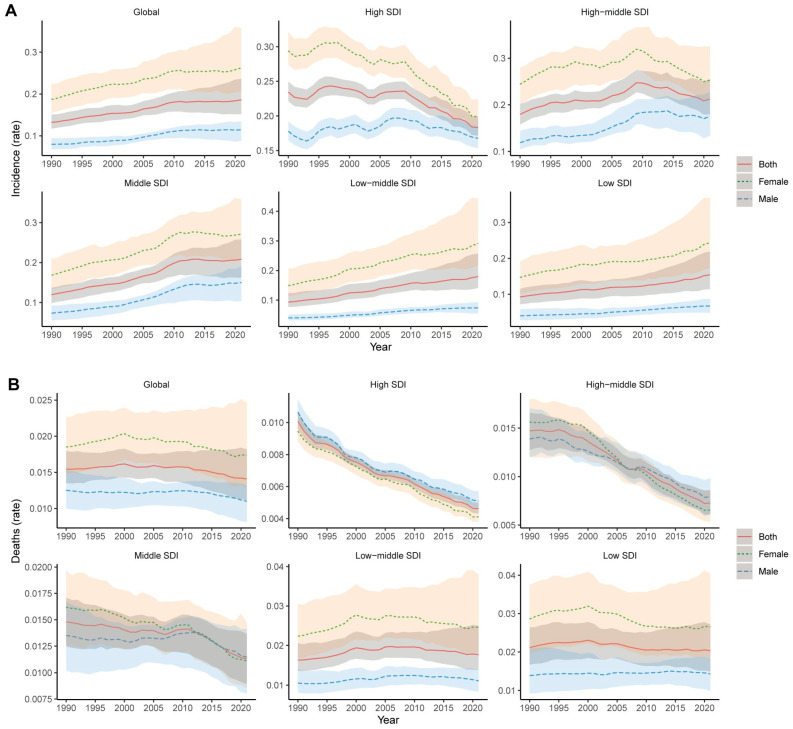
Trends in the incidence (**A**) and death (**B**) rates of thyroid cancer from 1990 to 2021 for males, females, and both across varying SDI levels. Reprinted/adapted with permission from Ref. [20]. 2024, Institute for Health Metrics and Evaluation.

We also present the incidence rates of thyroid cancer in children and adolescents from 1990 to 2021, stratified by SDI level and sex. Figure 3A demonstrates a global increase in incidence rates, with the steepest rises observed in low- and low–middle-SDI regions, while high-SDI regions show a plateau or decline in recent years. Females consistently exhibit higher incidence rates than males across all regions and SDI levels. Figure 3B shows a decline in global mortality rates, particularly in high-SDI and high–middle-SDI regions, where significant reductions are evident for both sexes. In contrast, the mortality rates in low- and low–middle-SDI regions remain stable or show slight increases. Notably, in high-SDI regions, males exhibit higher mortality rates than females, which contrasts with other SDI levels where females generally have higher mortality rates.

### 3.3. Age-Specific Trends in Thyroid Cancer Incidence and Mortality

**Figure 4:**
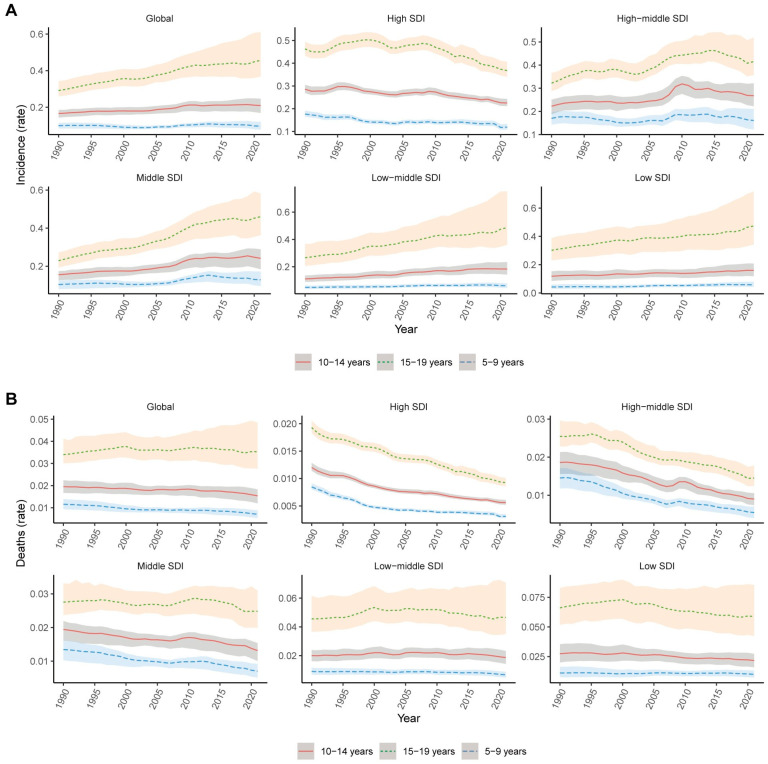
Trends in the incidence (**A**) and death (**B**) rates of thyroid cancer from 1990 to 2021 for different age groups (5–9 years old, 10–14 years old, 15–19 years old) across varying SDI levels. Reprinted/adapted with permission from Ref. [20]. 2024, Institute for Health Metrics and Evaluation.

We observed age-specific trends in thyroid cancer incidence and mortality among children and adolescents aged 5–9, 10–14, and 15–19 years old from 1990 to 2021. Figure 4A reveals a global increase in incidence rates across all SDI levels, with the highest rates consistently observed in the 15–19-years-old age group. The increase is most pronounced in low- and low–middle-SDI regions, while in high-SDI regions, incidence rates have plateaued or slightly declined in recent years. Figure 4B demonstrates a global decline in mortality rates, particularly in high-SDI and high–middle-SDI regions, where all age groups show decreasing trends. In contrast, mortality rates in low- and low–middle-SDI regions remain stable or show slight increases, with the 15–19-years-old group experiencing the highest mortality rates, while the 5–9-years-old group consistently has the lowest rates.

### 3.4. Sociodemographic Disparities in Incidence and Mortality Rates

**Figure 5:**
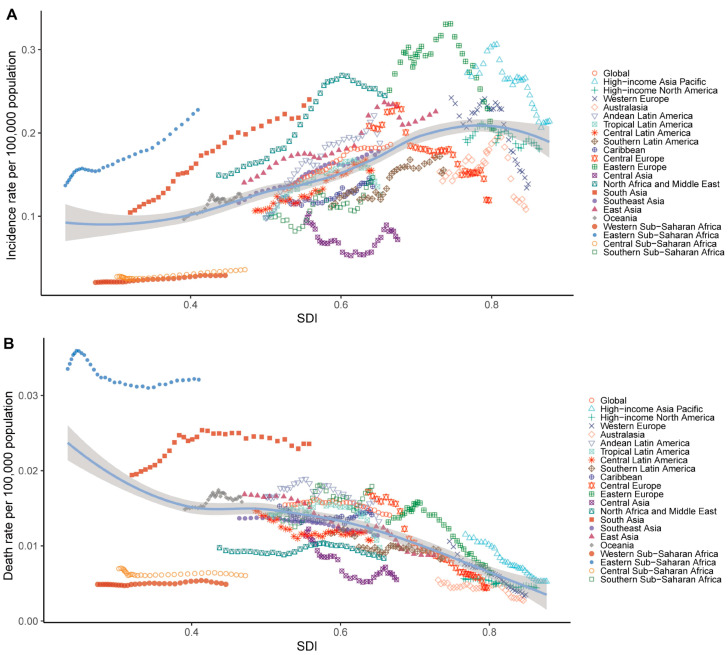
Relationship between the Sociodemographic Index (SDI) and thyroid cancer burden in children and adolescents in terms of incidence (**A**) and death (**B**) rate in 2021. Each point represents a region, with trends illustrating the correlation between the SDI and the respective rates. Reprinted/adapted with permission from Ref. [20]. 2024, Institute for Health Metrics and Evaluation.

We then illustrated the relationship between the SDI and both the incidence and death rates of the studied condition (per 100,000 population) across global regions. The incidence rate showed a significant positive correlation with the SDI (r = 0.593, *p* < 0.001). Higher-SDI regions, such as high-income Asian Pacific and Eastern Europe, exhibited the highest incidence rates. In contrast, regions with a lower SDI, such as Western Sub-Saharan Africa and South Asia, displayed markedly lower incidence rates (Figure 5A). In Figure 5B, the death rate demonstrated a significant negative correlation with the SDI (r = −0.495, *p* < 0.001). Regions with a lower SDI, particularly South Asian and Eastern Sub-Saharan Africa, exhibited relatively higher death rates, while higher-SDI regions displayed lower death rates.

### 3.5. Future Projections of Incidence and Mortality Rates

**Figure 6:**
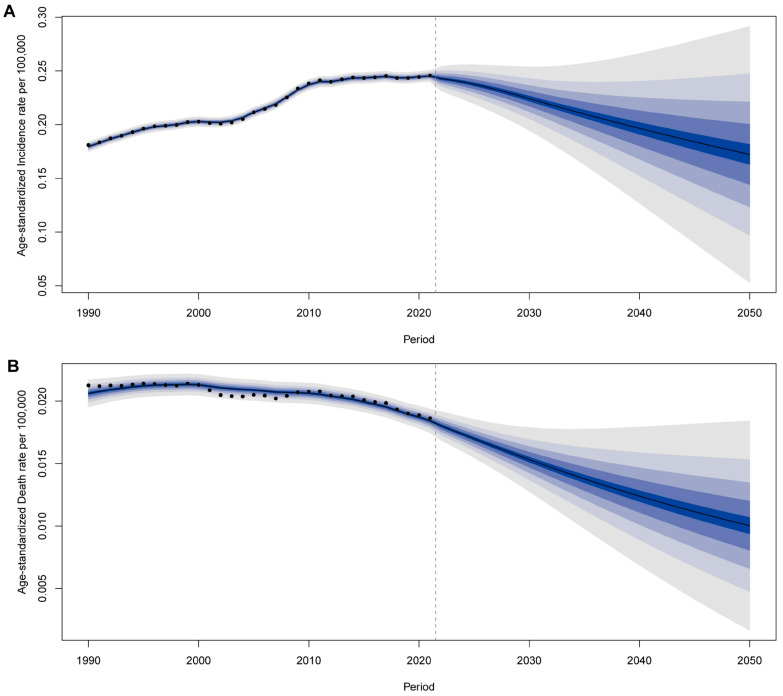
Projected trends in the age-standardized incidence rate (**A**) and death rate (**B**) of thyroid cancer in children and adolescents per 100,000 population from 2022 to 2050. Shaded areas represent the 95% uncertainty intervals for the projections. Reprinted/adapted with permission from Ref. [20]. 2024, Institute for Health Metrics and Evaluation.

We applied the Bayesian age–period–cohort (BAPC) model to estimate trends in age-standardized incidence and death rates per 100,000 population from 1990 to 2021 and projected rates through 2050. The incidence rate is projected to decline steadily from 2021 to 2050 (Figure 6A). Similarly, the death rate is expected to continue its downward trend, falling below 0.015 per 100,000 by 2050 (Figure 6B). The confidence intervals widen progressively, indicating increasing uncertainty in the long-term projections.

## 4. Discussion

Thyroid cancer in children and adolescents differs significantly from adult cases in molecular, pathological, and clinical aspects, often associated with a higher likelihood of lymph node metastasis and distant metastasis at the time of diagnosis, as well as increased postoperative recurrence rates [2,21]. The primary risk factors for thyroid cancer in children and adolescents include exposure to significant levels of ionizing radiation [22,23] and genetic predispositions [24]. Given the unique biological behavior, risk factors, and treatment responses, thyroid cancer in children and adolescents warrants focused research.

Our study revealed that the global incidence of thyroid cancer in children and adolescents increased significantly from 1990 to 2021. This trend was particularly pronounced in low-SDI regions such as South Asia, Andean Latin America, and North Africa, likely driven by improvements in healthcare systems, greater disease awareness, and broader availability of early screening programs [25,26]. However, overdiagnosis may also contribute to these trends in areas with increased healthcare access but limited diagnostic specificity [27]. In contrast, high-SDI regions, including Western Europe and high-income Asia-Pacific countries, showed stable or slightly declining incidence rates, possibly reflecting improved diagnostic protocols and reduced overdiagnosis. Notably, thyroid cancer in children and adolescents progresses more slowly and responds better to treatment compared to adults [28,29], underscoring the importance of targeted early detection and health education initiatives in high-incidence regions. The observed trends in thyroid cancer incidence may be influenced not only by actual changes in disease rates but also by advancements in diagnostic methods over time. In particular, improvements in diagnostic technologies, such as fine-needle aspiration biopsy [30,31], may have led to the increased detection of thyroid cancer. These advances could have contributed to the apparent rise in incidence, as more cases are diagnosed at an earlier stage.

It is noteworthy that while low-SDI regions have lower overall incidence rates, their rate of increase significantly outpaces that of high-income areas. This trend suggests that as economic development and awareness improve, there is an increasing ability to diagnose thyroid cancer, though the late detection of these cases remains a significant issue. Hence, early screening and public health education should become key priorities for intervention in these regions. It is important to note that thyroid cancer incidence may be underestimated, especially in low-SDI regions, due to lower diagnosis rates. Limited access to healthcare services in these regions can hinder early detection, contributing to this underreporting. Improving healthcare access and addressing health inequalities in low-SDI regions are crucial for more accurate assessments and better health outcomes.

Despite the rising incidence of thyroid cancer among children and adolescents, global mortality rates have modestly declined (EAPC: −0.27%), largely driven by improvements in high-SDI countries with advanced medical technologies and treatment options. In contrast, low-SDI regions, particularly South Asia, Southeast Asia, and Sub-Saharan Africa, continue to experience stable or rising mortality rates. These disparities highlight the urgent need to improve healthcare services, enhance early detection, and raise disease awareness in resource-limited regions to mitigate the burden of thyroid cancer [32]. While this study does not analyze the impact of different therapeutic strategies on mortality trends, it is important to consider how future research could integrate epidemiological modeling with treatment data. By incorporating data on therapeutic strategies and treatment outcomes, future studies could provide a more comprehensive understanding of declining mortality rates and regional disparities.

Our analysis also showed that age-specific differences further underscore the complexity of thyroid cancer in this population. Incidence rates were highest among adolescents aged 15–19 years old, surpassing rates in younger age groups. Adolescents face unique challenges, including psychosocial and physiological factors that may influence treatment adherence and outcomes [33,34]. Rapid hormonal changes during puberty, particularly fluctuations in estrogen [35,36] and growth hormone levels [37,38], may influence thyroid cell proliferation and increase susceptibility to malignancy. Additionally, the interplay between hormonal shifts and thyroid-stimulating hormone (TSH) levels during this critical developmental period could further impact cancer risk [39,40]. Tailored strategies, including psychological support, education on treatment adherence, and the development of less invasive treatments, are crucial for improving outcomes in adolescents. In low-SDI regions, where access to diagnosis and treatment may be limited, these challenges are further exacerbated, leading to poorer health outcomes. Health policies and access to treatment also play a key role in addressing health inequalities, especially in low-SDI regions. Limited healthcare access and inadequate infrastructure can delay diagnosis and treatment, worsening outcomes and widening disparities. Given these disparities, targeted public health interventions are needed in low-SDI regions to improve access to timely diagnosis, treatment, and care for adolescents. Addressing these gaps could significantly enhance the relevance of thyroid cancer control efforts and improve outcomes for this vulnerable population.

Based on the BAPC model predictions, the global incidence and mortality rates of thyroid cancer are expected to continue declining, particularly in high-SDI regions. However, trends in low-SDI regions may be constrained by disparities in healthcare resources, health education, and public health policies. Efforts to enhance early screening, optimize treatment strategies, and improve healthcare conditions remain key priorities for reducing the burden of thyroid cancer among children and adolescents globally.

Environmental factors play a significant role in the incidence and outcomes of thyroid cancer, and these effects should be explored in more detail. Several environmental factors, such as exposure to air pollution (e.g., fine particulate matter—PM2.5) [41], ionizing radiation [42,43], and dietary factors [44,45], have been linked to thyroid cancer risk. These factors vary across regions and may contribute to the observed regional disparities in thyroid cancer incidence and mortality. For example, regions with high levels of air pollution or those affected by nuclear accidents may experience higher rates of thyroid cancer, while dietary habits, such as iodine deficiency, may also play a role in certain areas.

This study was the first of its kind that provided an updated and comprehensive analysis of the global burden and trends of thyroid cancer among children and adolescents, emphasizing significant disparities in disease trends and outcomes among this unique population. However, several limitations should be acknowledged. First, this study relied on data from the Global Burden of Disease (GBD) database, which, despite its robustness, may be subject to inaccuracies due to the potential underreporting or misclassification of thyroid cancer cases. The database does not provide information on other medical conditions, health interventions, or the underreporting and misclassification of thyroid cancer cases, which prevents us from conducting sensitivity analyses to assess the potential impact of these factors on the study results. The misclassification and underreporting of thyroid cancer may affect epidemiological trends, but due to the lack of relevant data, we are unable to quantify the interference of these factors. Future research should incorporate additional data sources to assess these factors more comprehensively. Second, the lack of detailed individual-level data, such as genetic or environmental factors, limits the ability to explore specific risk factors or biological mechanisms underlying these trends. Third, while Bayesian age–period–cohort models are suitable for trend analysis, the accuracy of long-term projections may be limited in regions with rapidly evolving healthcare policies. The model assumes certain trends in healthcare access and policy, which may vary across regions, especially those undergoing rapid healthcare reforms. Regional differences in healthcare access and policy implementation can introduce uncertainty in the projections. Future research could incorporate dynamic models and regional-level data to improve the accuracy of long-term projections. Fourth, the uncertainty level of the projection results should be highlighted, especially for the long-term period (e.g., 2040–2050). The widening confidence intervals observed in these projections reflect the challenges of making long-term predictions. Lastly, the GBD database does not capture key environmental and behavioral risk factors for thyroid cancer, such as air pollution, ionizing radiation, and dietary factors. Moreover, it cannot account for individual-level factors like genetics or direct exposure to these risks. Future studies could integrate other data sources, such as cohort studies or cancer registries, to better assess these risk factors and improve the understanding of thyroid cancer’s etiology.

## 5. Conclusions

This study updates and expands the epidemiological understanding of the global burden and trends of thyroid cancer among children and adolescents, highlighting its rising incidence and declining mortality rates. Significant SDI-related health disparities were identified, and policymakers should focus on equitable resource allocation and develop targeted strategies to address these disparities, particularly in low-SDI regions and for vulnerable groups like adolescents aged 15–19, who exhibit the highest incidence and unique treatment challenges. Furthermore, to enhance the applicability and reliability of future research, it is crucial to improve data collection methods and integrate complementary methodological approaches, such as longitudinal studies and enhanced risk factor data. These improvements could help provide a more comprehensive understanding of thyroid cancer trends and inform healthcare policy and screening strategies to better meet the needs of at-risk populations.

## Figures and Tables

**Table 1:** Incidence of thyroid cancer in children and adolescents between 1990 and 2021 at the global and regional level.

	Incidence Cases, 1990	Incidence Rate Per 100,000 Population, 1990	Incidence Cases, 2021	Incidence Rate Per 100,000 Population, 2021	Estimated Annual Percentage Change, 1990–2021
Global	2981.399 (2646.628, 3405.199)	0.132 (0.117, 0.151)	4905.969 (3999.108, 6227.105)	0.186 (0.152, 0.236)	1.165 (1.058, 1.272)
Low SDI	258.561 (201.570, 322.236)	0.092 (0.072, 0.115)	900.696 (667.781, 1279.371)	0.154 (0.114, 0.219)	1.445 (1.369, 1.521)
Low–middle SDI	550.327 (454.377, 736.665)	0.093 (0.077, 0.125)	1372.150 (1078.872, 1969.170)	0.180 (0.141, 0.258)	2.187 (2.050, 2.323)
Middle SDI	915.357 (754.981, 1047.752)	0.120 (0.099, 0.137)	1560.292 (1213.828, 1932.164)	0.208 (0.162, 0.258)	2.028 (1.848, 2.208)
High–middle SDI	664.908 (583.870, 746.509)	0.180 (0.158, 0.202)	643.411 (544.860, 774.138)	0.212 (0.180, 0.255)	0.597 (0.365, 0.829)
High SDI	589.287 (556.073, 626.989)	0.234 (0.221, 0.249)	426.281 (398.648, 464.460)	0.183 (0.171, 0.200)	−0.689 (−0.890, −0.487)
Andean Latin America	18.577 (14.773, 24.044)	0.098 (0.078, 0.127)	44.509 (34.358, 58.246)	0.188 (0.145, 0.246)	2.488 (2.146, 2.831)
Australasia	9.621 (7.578, 12.153)	0.153 (0.121, 0.194)	8.170 (6.223, 10.694)	0.108 (0.083, 0.142)	−0.524 (−1.138, 0.093)
Caribbean	18.255 (14.951, 21.592)	0.121 (0.099, 0.143)	20.716 (15.590, 25.612)	0.136 (0.102, 0.168)	0.660 (0.508, 0.812)
Central Asia	29.238 (25.498, 33.481)	0.093 (0.081, 0.106)	24.933 (20.647, 29.671)	0.072 (0.060, 0.086)	−0.521 (−1.257, 0.221)
Central Europe	81.867 (73.240, 92.405)	0.208 (0.187, 0.235)	28.051 (24.788, 31.328)	0.119 (0.105, 0.133)	−1.675 (−1.932, −1.418)
Central Latin America	87.985 (81.912, 95.060)	0.106 (0.099, 0.115)	132.311 (116.216, 150.999)	0.155 (0.136, 0.177)	1.522 (1.373, 1.672)
Central Sub-Saharan Africa	8.526 (5.708, 12.714)	0.028 (0.018, 0.041)	26.417 (18.639, 38.909)	0.036 (0.025, 0.053)	1.075 (0.787, 1.363)
East Asia	645.674 (504.992, 755.266)	0.140 (0.110, 0.164)	777.893 (578.634, 989.299)	0.226 (0.168, 0.287)	1.568 (1.307, 1.830)
Eastern Europe	169.055 (155.225, 184.356)	0.251 (0.231, 0.274)	94.907 (87.765, 103.879)	0.206 (0.190, 0.225)	−0.701 (−1.128, −0.272)
Eastern Sub-Saharan Africa	151.859 (116.920, 194.851)	0.137 (0.105, 0.176)	518.339 (365.876, 800.784)	0.228 (0.161, 0.352)	1.416 (1.256, 1.577)
High-income Asia Pacific	136.937 (117.871, 164.941)	0.272 (0.234, 0.328)	65.901 (56.307, 78.852)	0.214 (0.183, 0.256)	−1.084 (−1.311, −0.856)
High-income North America	157.131 (150.216, 164.814)	0.192 (0.184, 0.202)	161.720 (152.518, 171.810)	0.181 (0.170, 0.192)	−0.322 (−0.470, −0.175)
North Africa and Middle East	264.413 (203.685, 378.633)	0.150 (0.115, 0.214)	579.403 (476.164, 713.265)	0.245 (0.201, 0.302)	2.170 (1.856, 2.484)
Oceania	3.241 (2.130, 4.440)	0.096 (0.063, 0.132)	7.846 (4.640, 11.452)	0.123 (0.073, 0.179)	0.685 (0.499, 0.871)
South Asia	566.810 (455.956, 749.749)	0.104 (0.084, 0.138)	1641.728 (1215.304, 2425.352)	0.240 (0.178, 0.355)	2.736 (2.579, 2.894)
Southeast Asia	255.818 (184.743, 306.437)	0.116 (0.084, 0.139)	407.968 (305.445, 515.382)	0.178 (0.133, 0.225)	1.286 (1.202, 1.370)
Southern Latin America	21.758 (18.145, 25.820)	0.112 (0.094, 0.133)	30.346 (24.708, 37.096)	0.156 (0.127, 0.190)	1.294 (1.057, 1.533)
Southern Sub-Saharan Africa	26.156 (19.847, 32.112)	0.099 (0.075, 0.121)	45.835 (35.215, 59.725)	0.147 (0.113, 0.191)	1.212 (0.813, 1.612)
Tropical Latin America	68.083 (61.868, 74.324)	0.098 (0.089, 0.107)	83.954 (74.671, 94.132)	0.126 (0.112, 0.141)	1.264 (0.849, 1.680)
Western Europe	238.222 (220.235, 258.696)	0.242 (0.224, 0.263)	127.192 (114.555, 142.860)	0.139 (0.125, 0.156)	−1.525 (−1.948, −1.100)
Western Sub-Saharan Africa	22.173 (16.219, 28.565)	0.021 (0.015, 0.027)	77.830 (50.505, 108.567)	0.029 (0.019, 0.040)	1.300 (1.184, 1.415)

**Table 2:** Death from thyroid cancer in children and adolescents between 1990 and 2021 at the global and regional level.

	Death Cases, 1990	Death Rate Per 100,000 Population, 1990	Death cases, 2021	Death Rate Per 100,000 Population, 2021	Estimated Annual Percentage Change, 1990–2021
Global	348.775 (305.523, 405.949)	0.015 (0.014, 0.018)	371.642 (294.203, 479.622)	0.014 (0.011, 0.018)	−0.272 (−0.378, −0.166)
Low SDI	59.030 (46.834, 73.813)	0.021 (0.017, 0.026)	118.828 (87.431, 158.709)	0.020 (0.015, 0.027)	−0.351 (−0.454, −0.248)
Low–middle SDI	96.387 (80.681, 121.561)	0.016 (0.014, 0.021)	135.161 (105.958, 190.458)	0.018 (0.014, 0.025)	0.304 (0.096, 0.513)
Middle SDI	113.218 (95.339, 130.713)	0.015 (0.012, 0.017)	84.566 (66.923, 102.288)	0.011 (0.009, 0.014)	−0.690 (−0.841, −0.539)
High–middle SDI	54.505 (47.147, 61.431)	0.015 (0.013, 0.017)	22.083 (18.521, 26.151)	0.007 (0.006, 0.009)	−2.491 (−2.675, −2.306)
High SDI	25.331 (24.138, 26.874)	0.010 (0.010, 0.011)	10.749 (10.043, 11.620)	0.005 (0.004, 0.005)	−2.318 (−2.391, −2.245)
Andean Latin America	3.149 (2.685, 3.867)	0.017 (0.014, 0.020)	3.141 (2.526, 3.960)	0.013 (0.011, 0.017)	−0.466 (−0.735, −0.195)
Australasia	0.343 (0.293, 0.403)	0.005 (0.005, 0.006)	0.207 (0.171, 0.256)	0.003 (0.002, 0.003)	−1.524 (−1.977, −1.069)
Caribbean	2.307 (1.747, 2.854)	0.015 (0.012, 0.019)	2.159 (1.401, 2.837)	0.014 (0.009, 0.019)	−0.019 (−0.172, 0.135)
Central Asia	3.572 (3.292, 3.889)	0.011 (0.010, 0.012)	1.903 (1.641, 2.217)	0.005 (0.005, 0.006)	−2.367 (−2.934, −1.797)
Central Europe	6.735 (6.266, 7.196)	0.017 (0.016, 0.018)	1.040 (0.940, 1.145)	0.004 (0.004, 0.005)	−4.279 (−4.544, −4.014)
Central Latin America	12.134 (11.543, 12.783)	0.015 (0.014, 0.015)	9.142 (8.124, 10.315)	0.011 (0.010, 0.012)	−0.684 (−0.910, −0.459)
Central Sub-Saharan Africa	2.155 (1.430, 3.183)	0.007 (0.005, 0.010)	4.441 (3.200, 6.136)	0.006 (0.004, 0.008)	−0.253 (−0.399, −0.106)
East Asia	77.521 (60.060, 91.935)	0.017 (0.013, 0.020)	30.135 (21.303, 38.525)	0.009 (0.006, 0.011)	−2.303 (−2.505, −2.101)
Eastern Europe	8.851 (8.292, 9.416)	0.013 (0.012, 0.014)	2.974 (2.763, 3.234)	0.006 (0.006, 0.007)	−2.773 (−3.316, −2.226)
Eastern Sub-Saharan Africa	37.156 (29.041, 47.950)	0.034 (0.026, 0.043)	73.024 (51.538, 105.177)	0.032 (0.023, 0.046)	−0.420 (−0.539, −0.300)
High-income Asia Pacific	5.793 (4.995, 7.048)	0.012 (0.010, 0.014)	1.620 (1.455, 1.923)	0.005 (0.005, 0.006)	−2.700 (−2.836, −2.564)
High-income North America	4.612 (4.478, 4.758)	0.006 (0.005, 0.006)	3.925 (3.715, 4.139)	0.004 (0.004, 0.005)	−0.913 (−1.031, −0.795)
North Africa and Middle East	17.250 (13.202, 27.073)	0.010 (0.007, 0.015)	19.746 (16.220, 23.685)	0.008 (0.007, 0.010)	−0.005 (−0.239, 0.229)
Oceania	0.500 (0.332, 0.704)	0.015 (0.010, 0.021)	1.012 (0.598, 1.471)	0.016 (0.009, 0.023)	0.299 (0.147, 0.452)
South Asia	105.341 (87.265, 135.119)	0.019 (0.016, 0.025)	160.989 (121.138, 230.151)	0.024 (0.018, 0.034)	0.596 (0.351, 0.841)
Southeast Asia	30.048 (22.272, 35.839)	0.014 (0.010, 0.016)	26.101 (20.876, 30.454)	0.011 (0.009, 0.013)	−0.590 (−0.659, −0.521)
Southern Latin America	2.259 (2.004, 2.543)	0.012 (0.010, 0.013)	1.636 (1.374, 1.918)	0.008 (0.007, 0.010)	−0.841 (−1.102, −0.580)
Southern Sub-Saharan Africa	3.739 (3.005, 4.485)	0.014 (0.011, 0.017)	5.605 (4.386, 7.148)	0.018 (0.014, 0.023)	0.769 (0.439, 1.100)
Tropical Latin America	9.644 (8.844, 10.491)	0.014 (0.013, 0.015)	6.509 (5.788, 7.269)	0.010 (0.009, 0.011)	−0.535 (−0.918, −0.150)
Western Europe	10.426 (9.956, 10.915)	0.011 (0.010, 0.011)	3.207 (2.930, 3.555)	0.003 (0.003, 0.004)	−3.125 (−3.368, −2.882)
Western Sub-Saharan Africa	5.241 (3.929, 6.682)	0.005 (0.004, 0.006)	13.126 (8.529, 17.681)	0.005 (0.003, 0.007)	0.278 (0.178, 0.378)

## Data Availability

Data are contained within the article.

## Abbreviations

GBD	Global Burden of Disease
EAPC	Estimated annual percentage change
SDI	Sociodemographic Index
PTC	Papillary thyroid carcinoma
ASR	Age-standardized rate
BAPC	Bayesian age–period–cohort model
RLN	Recurrent Laryngeal Nerve
